# Supplementary material for: Atmospheric biogenic volatile organic compounds in the Alaskan Arctic tundra: constraints from measurements at Toolik Field Station
Source: Atmos Chem Phys. Author manuscript; Available in PMC 2023 Jul 20. (PMC10358744; doi:10.5194/acp-22-14037-2022)
Supplement: Supplementary material [file NIHMS1864742-supplement-Supplementary_material.zip › SupplementalFigures_Toolik.docx]

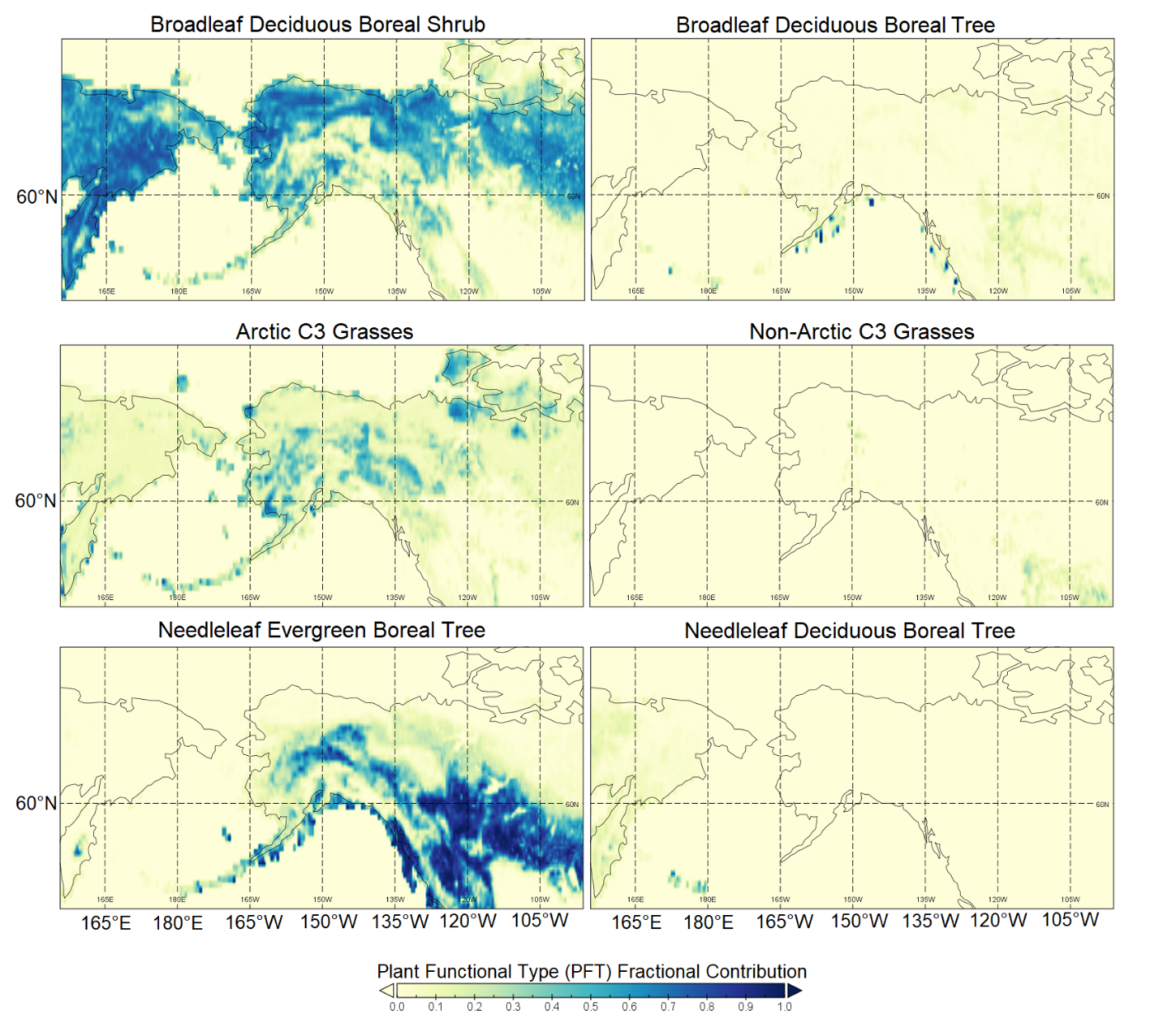


**Figure S1.** Predominant plant functional types (PFTs) occurring in Alaska shown as their fractional contribution (0 to 1). PFTs not shown (9) are not within the Alaskan domain. PFTs come from Community Land Map 4 (CLM4), and Guenther et al., 2012.


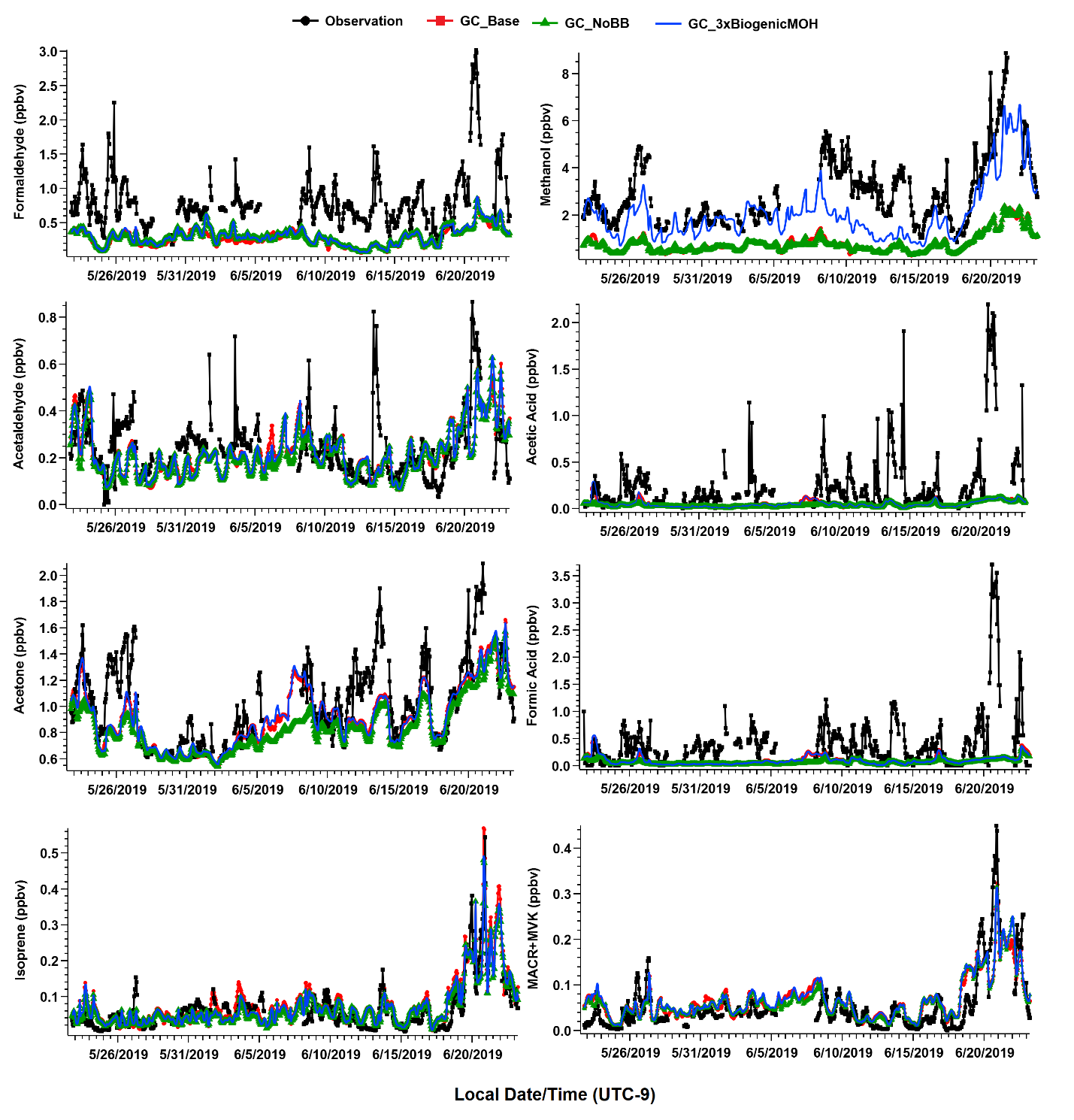


**Figure S2.** Ambient mixing ratios (ppbv) of observed (black) and GEOS-Chem+MEGANv2.1 simulated (GC_Base; red) VOCs. Simulations are also shown for GOES-Chem+MEGANv2.1 simulations with biomass burning emissions removed (GC_NoBB; green) and with direct biogenic emissions of methanol increased by a factor of three (GC_3×BiogenicMOH; blue).


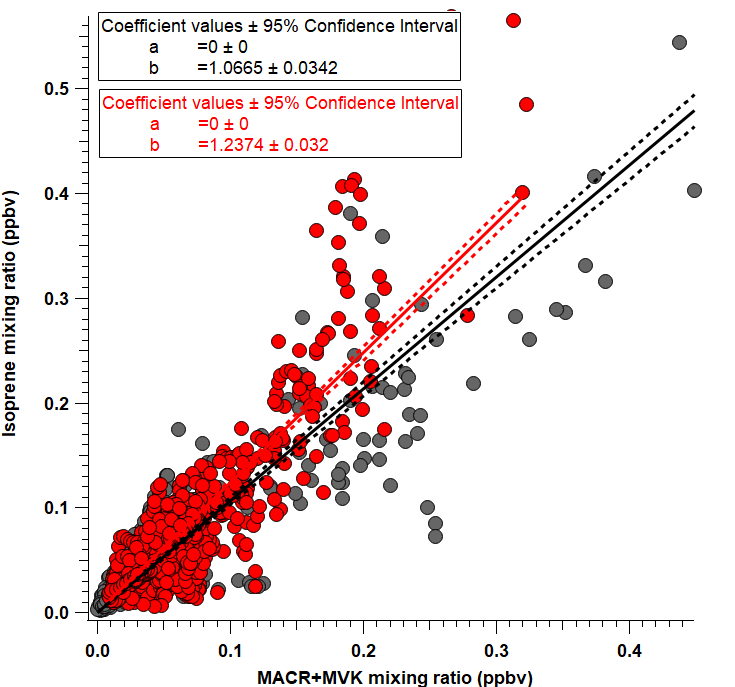


**Figure S3.** Ambient mixing ratios (ppbv) of observed (black) and GEOS-Chem+MEGANv2.1 simulated (red) isoprene versus MACR+MVK. Fit shown is major axis regression, with intercept forced through zero. Equation is in the form of *y = bx + a.*

**
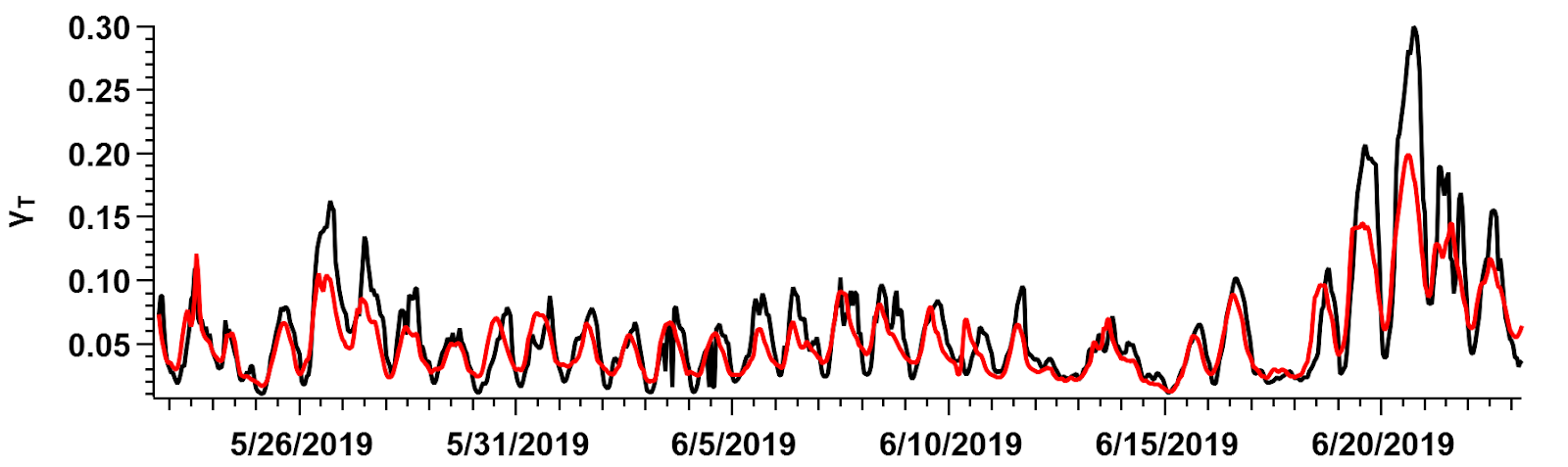
**

**Figure S4.** Temperature activity factor (γ_T_) for isoprene computed based on observed and simulated surface air temperature at TFS. Values in black are based on observed 3 m air temperature from the meteorological tower at TFS, and values in red are based on assimilated 2 m air temperature from the GEOS-FP meteorological field.


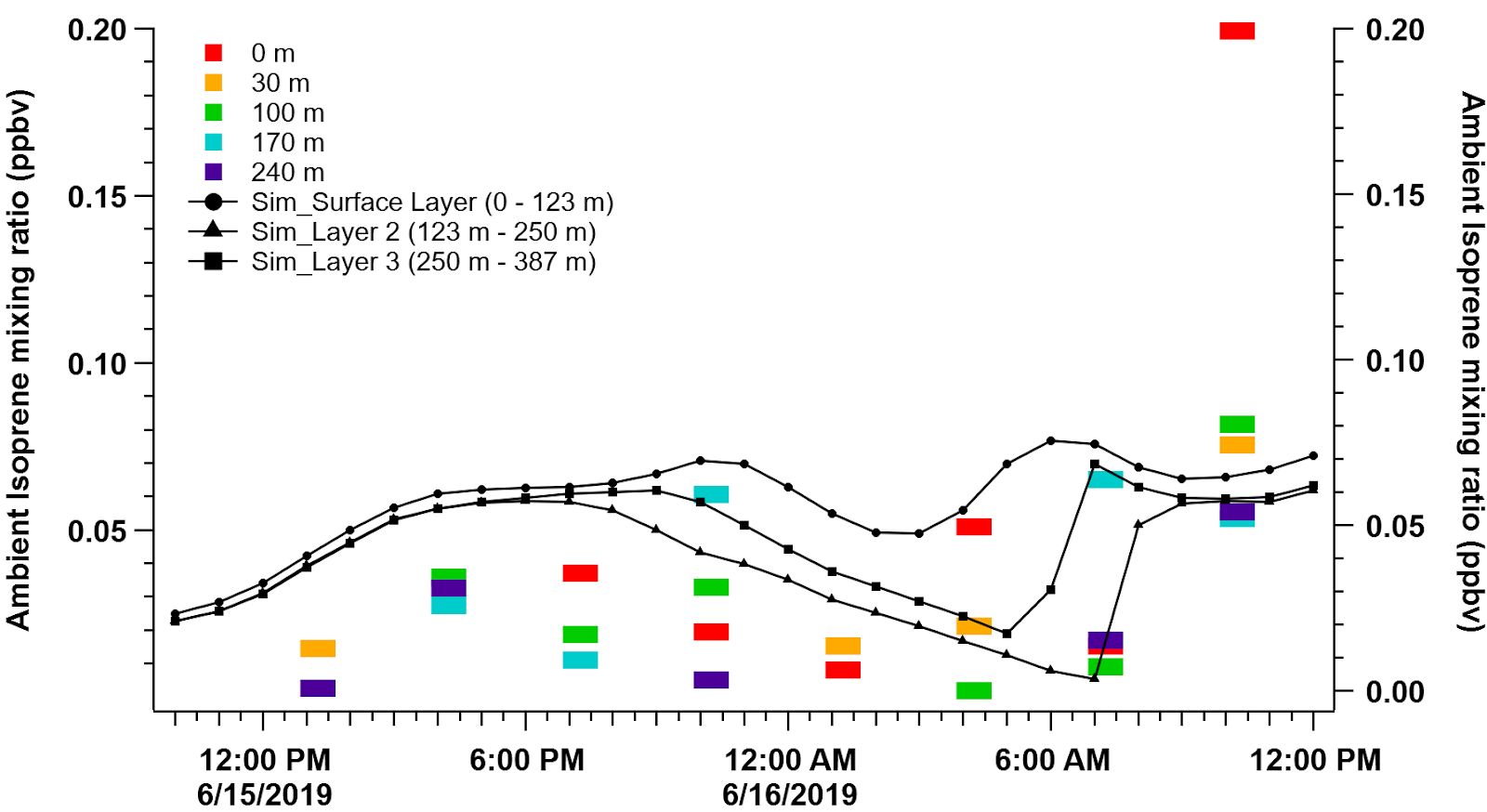


**Figure S5.** Vertical profiles of ambient isoprene mixing ratios collected from 30 min tethered balloon samples (see Angot et al., 2020), and simulated isoprene mixing ratio within the first three layers (surface to ~350m above ground level) of GEOS-Chem.


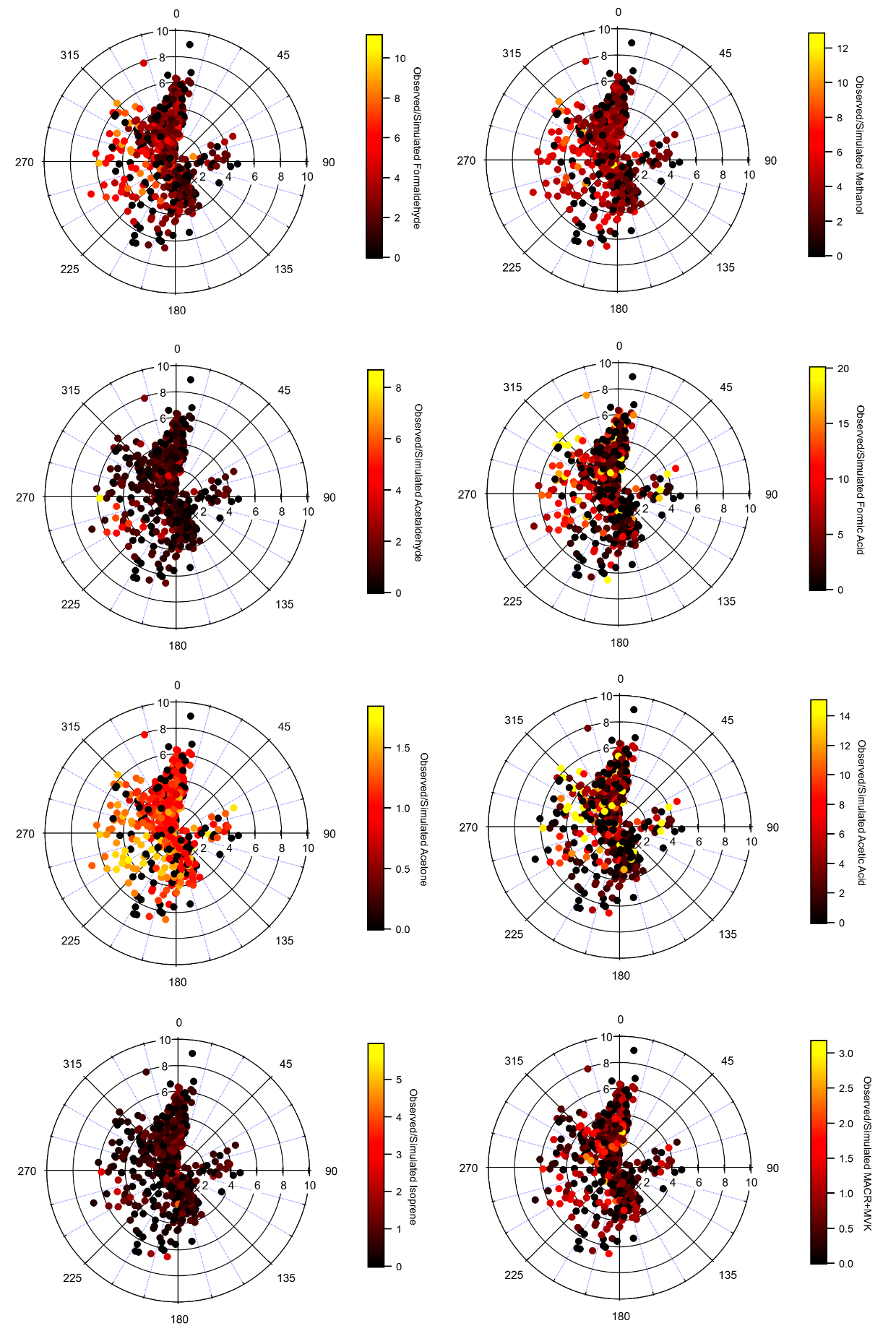


**Figure S6.** Polar wind plots of hourly wind speed, direction, and ratio between observed and simulated VOC mixing ratio (color bar). Note that each VOC species has a different scale.


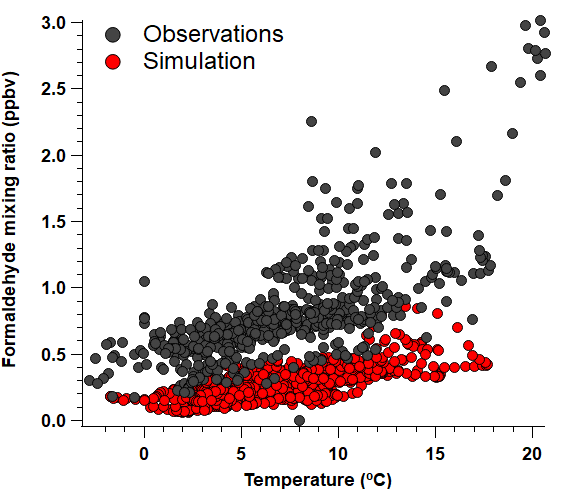


**Figure S7.** Ambient mixing ratios (ppbv) of observed (black) and GEOS-Chem+MEGANv2.1 simulated (red) formaldehyde versus corresponding observed and assimilated temperature (r^2^ = >0.5).
