# Supplementary material for: Atmospheric biogenic volatile organic compounds in the Alaskan Arctic tundra: constraints from measurements at Toolik Field Station
Source: Atmos Chem Phys. Author manuscript; Available in PMC 2023 Jul 20. (PMC10358744; doi:10.5194/acp-22-14037-2022)
Supplement: Supplementary material [file NIHMS1864742-supplement-Supplementary_material.zip › acp-22-14037-2022-supplement-title-page.pdf]

Supplement of Atmos. Chem. Phys., 22, 14037–14058, 2022
https://doi.org/10.5194/acp-22-14037-2022-supplement
© Author(s) 2022. CC BY 4.0 License.

Supplement of

Atmospheric biogenic volatile organic compounds in the Alaskan Arctic tundra: constraints from measurements at Toolik Field Station

Vanessa Selimovic et al.

Correspondence to: Lu Hu (lu.hu@mso.umt.edu) and Vanessa Selimovic (vanessa.selimovic@umontana.edu)

- acp-22-14037-2022-supplement-title-page.pdf
- SuppTable2.xlsx
- SupplementalFigures_Toolik.docx

The copyright of individual parts of the supplement might differ from the article licence.
